# Manganese Uptake, Mediated by SloABC and MntH, Is Essential for the Fitness of Streptococcus mutans

**DOI:** 10.1128/mSphere.00764-19

**Published:** 2020-01-08

**Authors:** Jessica K. Kajfasz, Callahan Katrak, Tridib Ganguly, Jonathan Vargas, Logan Wright, Zachary T. Peters, Grace A. Spatafora, Jacqueline Abranches, José A. Lemos

**Affiliations:** a Department of Oral Biology, University of Florida College of Dentistry, Gainesville, Florida, USA; b Department of Biology, Middlebury College, Middlebury, Vermont, USA; University of Kentucky

**Keywords:** *S. mutans*, manganese, metal transport, stress response, dental caries, biofilm, *Streptococcus mutans*

## Abstract

Early epidemiological studies implicated manganese (Mn) as a possible caries-promoting agent, while laboratory studies have indicated that manganese stimulates the expression of virulence-related factors in the dental pathogen Streptococcus mutans. To better understand the importance of manganese homeostasis to S. mutans pathophysiology, we first used RNA sequencing to obtain the global transcriptional profile of S. mutans UA159 grown under Mn-restricted conditions. Among the most highly expressed genes were those of the entire *sloABC* operon, encoding a dual iron/manganese transporter, and an uncharacterized gene, here *mntH*, that codes for a protein bearing strong similarity to Nramp-type transporters. While inactivation of *sloC*, which encodes the lipoprotein receptor of the SloABC system, or of *mntH* alone had no major consequence for the overall fitness of S. mutans, simultaneous inactivation of *sloC* and *mntH* (Δ*sloC* Δ*mntH*) impaired growth and survival under Mn-restricted conditions, including in human saliva or in the presence of calprotectin. Further, disruption of Mn transport resulted in diminished stress tolerance and reduced biofilm formation in the presence of sucrose. These phenotypes were markedly improved when cells were provided with excess Mn. Metal quantifications revealed that the single mutant strains contained intracellular levels of Mn similar to those seen with the parent strain, whereas Mn was nearly undetectable in the Δ*sloC* Δ*mntH* strain. Collectively, these results reveal that SloABC and MntH work independently and cooperatively to promote cell growth under Mn-restricted conditions and that maintenance of Mn homeostasis is essential for the expression of major virulence attributes in S. mutans.

**IMPORTANCE** As transition biometals such as manganese (Mn) are essential for all forms of life, the ability to scavenge biometals in the metal-restricted host environment is an important trait of successful cariogenic pathobionts. Here, we showed that the caries pathogen Streptococcus mutans utilizes two Mn transport systems, namely, SloABC and MntH, to acquire Mn from the environment and that the ability to maintain the cellular levels of Mn is important for the manifestation of characteristics that associate S. mutans with dental caries. Our results indicate that the development of strategies to deprive S. mutans of Mn hold promise in the combat against this important bacterial pathogen.

## Abstract (precis)

As transition biometals such as manganese (Mn) are essential for all forms of life, the ability to scavenge biometals in the metal-restricted host environment is an important trait of successful cariogenic pathobionts. Here, we showed that the caries pathogen Streptococcus mutans utilizes two Mn transport systems, namely, SloABC and MntH, to acquire Mn from the environment and that the ability to maintain the cellular levels of Mn is important for the manifestation of characteristics that associate S. mutans with dental caries. Our results indicate that the development of strategies to deprive S. mutans of Mn hold promise in the combat against this important bacterial pathogen.

## INTRODUCTION

Transition metals are essential for all domains of life by serving as structural and catalytic cofactors, with approximately 50% of all enzymes in cells requiring a metal cofactor for proper function (1). During microbial infections, the ability of the invading pathogen to acquire iron (Fe), manganese (Mn), and zinc (Zn) becomes particularly relevant as the host employs several mechanisms to sequester these essential biometals as part of an active response known as nutritional immunity (2–5). Specifically, Fe-binding proteins such as transferrin (in serum) and lactoferrin (in secretions) are produced by the host to chelate Fe, thereby restricting its bioavailability to invading pathogens. Similarly, transition metals are actively sequestered by calprotectin, a heterodimeric S100 family protein that is an important part of the inflammatory response during infection, was named for its role in innate immunity, and constitutes about 60% of the total proteins in neutrophils (3, 6, 7). To overcome this micronutrient limitation, bacteria evolved a number of mechanisms for metal acquisition, including the production of low-molecular-weight molecules (metallophores) for extracellular metal capture and of high-affinity membrane-associated metal transporters, as well as tools for direct acquisition of metal from host molecules and proteins (metal piracy) (5).

Streptococcus mutans is regarded as a keystone pathogen in dental caries due to its ability to change the architecture and environment of oral biofilm such that it fosters the outgrowth of acidogenic and aciduric species (such as *Lactobacillus* spp., *Actinomyces* spp., *Bifidobacterium* spp., Scardovia wiggsiae, Streptococcus sobrinus, and S. mutans itself) at the expense of the commensal bacteria associated with oral health (8, 9). The cariogenic potential of S. mutans resides in its ability to (i) form robust biofilms on tooth surfaces in a sucrose-dependent manner; (ii) produce and tolerate large amounts of lactic acid, the major end product of its fermentative metabolism; and (iii) cope with the oxidative stress that arises from the environmental reduction of oxygen and the production of hydrogen peroxide (H_2_O_2_) by competing neighbor species (10). In addition to dental caries, S. mutans is also one of the causative agents of infective endocarditis, a life-threatening bacterial infection of the endocardium (11).

Previous studies conducted during the 1970s and 1980s indicated a possible relationship between biometal availability in the oral cavity and caries incidence (12–16). In particular, high rates of caries were linked to elevated levels of Mn in drinking water (12, 14, 16). Despite the existence of conflicting clinical data questioning this correlation (13, 15), few studies have directly investigated the significance of Mn in the pathophysiology of oral streptococci (17–25). An early study aiming to determine the trace element requirement of oral streptococci concluded that Mn was the only trace metal absolutely required for the growth of cariogenic and noncariogenic streptococci in the laboratory setting (22), a finding that was later confirmed by a second group of investigators (17). In addition, Mn was shown to stimulate dextran-dependent aggregation in Streptococcus criceti (formerly S. cricetus) (26), a trait that was found to be mediated by surface-associated glucan-binding proteins (GBPs) and to be critical to sucrose-dependent adhesion and biofilm formation (27). Subsequent studies using both S. criceti and Streptococcus sobrinus strains showed that metal chelating agents such as citrate or EDTA reversibly inhibit glucan-induced aggregation, thereby preventing sucrose-dependent adhesion (24). In addition, confocal microscopy analysis of S. mutans UA159 biofilms grown in the presence of sucrose revealed that Mn-depleted biofilms formed large cell clumps that were more easily washed away than biofilms formed under Mn-replete conditions (18). Manganese was also shown to stimulate carbohydrate metabolism in S. mutans, in particular, the synthesis of glycogen-like intracellular polysaccharide (IPS) stores (21). Finally, when added to drinking water, Mn was shown to increase the cariogenic potential of S. mutans in a germfree rat model (21). It should also be noted that Mn is known to play an important role in the oxidative stress responses of lactic acid bacteria by directly interacting with and scavenging superoxide radicals, by serving as the enzymatic cofactor of the superoxide dismutase (SOD) enzyme, and by replacing Fe as an enzymatic cofactor, thereby protecting Fe-binding proteins from the irreversible damage of Fenton chemistry (28, 29). Collectively, the picture that emerges from these studies is that Mn may serve as a caries-promoting agent by stimulating bacterial metabolism, by facilitating sucrose-dependent biofilm formation, and possibly by conferring protection against the oxidative stresses encountered in dental plaque.

Because the nutrients available in the oral cavity derive, in large part, from the diet, the concentration of Mn in human saliva has been shown to fluctuate from as low as 1 μM (13, 15) to as high as 36 μM (30). Taking into consideration that the concentration of Mn is restricted to the nanomolar range in plasma (31), the concentration of Mn in saliva is unlikely to be a growth-limiting factor for most oral bacteria. And yet, fluctuations in Mn levels may serve as a cue for S. mutans to sense the environment and adjust its metabolism accordingly by favoring a biofilm survival mode over an active-growth mode and/or dispersion mode. Beyond the oral environment, the ability to scavenge Mn in environments in which availability of this metal is known to be restricted, such as the bloodstream and internal organs, has proven to be an essential trait for bacterial pathogens. In fact, a growing number of Mn transport systems have been identified as major virulence factors, including examples where loss of Mn transporters rendered organisms closely related to S. mutans, such as Streptococcus pneumoniae and Enterococcus faecalis, virtually avirulent in animal infection models (32, 33). In S. mutans, previous characterization of pathways associated with Mn homeostasis has been restricted to the metalloregulator SloR and the ABC-type transporter SloABC (34–38). Those studies revealed that specific binding to Fe or Mn triggered function of SloR as a global transcriptional repressor, which includes repression of the *sloABC* operon (35–37). SloABC was shown to function as a dual Fe and Mn transporter, and the virulence of a *sloA* mutant strain was attenuated in a rat model of endocarditis (38).

To further our understanding of the significance of Mn homeostasis for S. mutans pathobiology, we first used RNA deep sequencing (RNA-Seq) to compare the transcriptomes of S. mutans serotype *c* strain UA159 grown in a chemically defined medium under Mn-depleted and Mn-replete conditions. Among the genes highly upregulated during Mn starvation were all genes of the *sloABC* operon and S. mutans *770c* (*smu770c*), here *mntH*, coding for a putative metal transporter from the natural resistance-associated macrophage protein-type (Nramp) family. While inactivation of *sloC*, coding for the SloC lipoprotein receptor, or of *mntH* alone did not cause a significant impact in the overall fitness of S. mutans, simultaneous inactivation of *sloC* and *mntH* (Δ*sloC* Δ*mntH* strain) resulted in a dramatic reduction in cellular Mn levels and impaired growth and survival when cells were grown under Mn-restricted conditions. Further characterization of the Δ*sloC*, Δ*mntH*, and Δ*sloC* Δ*mntH* strains revealed that Mn transport contributes to the ability *of* S. mutans to cope with acid and oxidative stresses and to form biofilms in the presence of sucrose. Collectively, the data from this study reveal that Mn transport in S. mutans is primarily mediated by SloABC and MntH and support the idea that Mn plays a critical role in the expression of virulence attributes by this important human pathogen.

## RESULTS

### Transcriptome analysis reveals a new Mn transporter in S. mutans.

**FIG 1:**
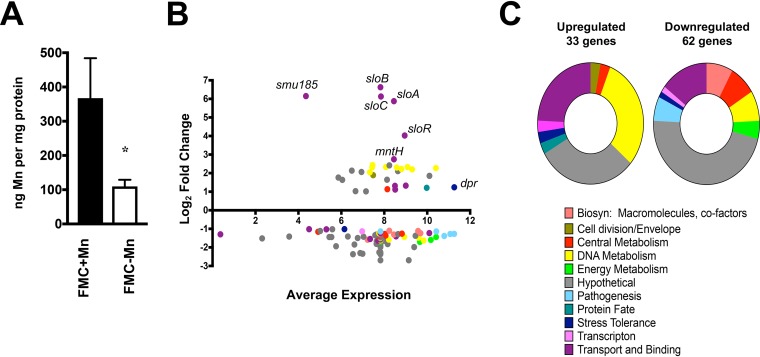
Summary of RNA-Seq analysis comparing S. mutans UA159 grown under Mn-depleted versus Mn-replete conditions. S. mutans UA159 was grown to an OD_600_ of 0.4 in FMC medium (complete or depleted of Mn). Total RNA was isolated, and the levels of gene expression under each condition were compared via RNA-Seq analysis. (A) Intracellular Mn content of S. mutans UA159 grown to an OD_600_ of ∼0.4 in FMC medium (complete or depleted of Mn). The bar graphs show averages and standard deviations of results from five independent ICP-OES analyses. Student's *t* test was used to compare levels of metal content between the two media (*, *P ≤ *0.005). (B) Dot plot of genes differentially expressed under conditions of Mn depletion as determined by Degust (degust.erc.monash.edu). The *y* axis indicates the log_2_ fold change in expression compared to control cultures (FMC complete), while the *x* axis indicates the average expression level of each gene compared to all other genes. The identities of selected genes of interest are indicated. (C) Graphical representations of the functional categories for upregulated or downregulated genes shown in panel B. Biosyn, biosynthesis.

Comparison of the transcriptome profiles of UA159 grown to mid-exponential phase in a chemically defined medium depleted for Mn (∼0.2 μM Mn) versus growth under Mn-replete (∼130 μM Mn) conditions identified 95 differentially expressed genes (Table 1) (false-discovery rate [FDR] of 0.01, 2-fold cutoff). Among those, 33 genes were upregulated and 62 were downregulated. To ensure that these gene expression trends were indeed due to Mn restriction, the intracellular Mn content of S. mutans UA159 grown under Mn-replete or Mn-depleted conditions was determined using inductively coupled plasma optical emission spectrometry (ICP-OES). The analysis confirmed that intracellular Mn content was severely diminished when S. mutans UA159 was grown in the Mn-depleted FMC medium (Fig. 1A).

**TABLE 1:** S. mutans genes differentially expressed when grown in FMC depleted of Mn compared to FMC complete media

Locus	Gene name, function	Fold change	*P* value
Upregulated			
SMU_0082	*dnaK*, chaperone protein	2.31	7.03E−06
SMU_0182	*sloA*, ABC transporter, ATP-binding protein	58.87	5.58E−16
SMU_0183	*sloB*, ABC transporter permease element	99.02	1.34E−16
SMU_0184	*sloC*, ABC transporter, substrate binding protein	70.07	1.43E−17
SMU_0185	Hypothetical protein	71.05	4.86E−13
SMU_0186	*sloR*, metal-dependent transcriptional regulator	16.30	1.73E−16
SMU_0438c	(R)-2-hydroxyglutaryl-CoA dehydratase activator-related protein^*a*^	2.20	2.27E−04
SMU_0503c	Hypothetical protein	3.39	9.07E−09
SMU_0540	*dpr*, peroxide resistance protein/iron binding protein	2.36	3.82E−07
SMU_0600c	Conserved hypothetical protein	2.04	4.46E−06
SMU_0609	*bsp*, cell wall protein precursor	3.71	4.34E−11
SMU_0635	Conserved hypothetical protein	4.32	4.98E−12
SMU_0768c	Conserved hypothetical protein	4.40	2.97E−11
SMU_0769	Conserved hypothetical protein	2.03	1.68E−10
SMU_0770c	*mntH*, manganese transporter periplasmic protein	6.73	2.52E−13
SMU_0941c	Conserved hypothetical protein	3.13	7.44E−06
SMU_0984	Hypothetical protein	3.12	1.07E−08
SMU_0996	*yclN*, ABC transporter, permease protein	2.17	2.27E−03
SMU_0997	*fecE*, ABC transporter, ATP-binding protein	2.49	9.29E−04
SMU_0998	*fatB*, ABC transporter, ferrichrome-binding protein	2.51	5.60E−04
SMU_1750c	Hypothetical protein	4.19	1.42E−09
SMU_1752c	Hypothetical protein	3.62	3.21E−08
SMU_1753c	CRISPR2-Cas	5.04	2.32E−10
SMU_1754c	CRISPR2-Cas	5.35	4.14E−10
SMU_1755c	CRISPR2-Cas	4.99	2.58E−10
SMU_1757c	CRISPR2-Cas	5.41	6.85E−10
SMU_1758c	CRISPR2-Cas	4.94	1.15E−10
SMU_1760c	CRISPR2-Cas	5.03	2.32E−10
SMU_1761c	CRISPR2-Cas	4.61	2.60E−10
SMU_1762c	CRISPR2-Cas	4.13	4.47E−10
SMU_1763c	CRISPR2-Cas	4.68	6.72E−10
SMU_1764c	CRISPR2-Cas	4.84	2.33E−10
SMU_2027	Transcriptional regulator/repressor	2.17	9.31E−08

Downregulated			
SMU_0029	*purC*, phosphoribosylaminoimidazole-succinocarboxamide synthase	−3.00	3.37E−08
SMU_0030	*purL*, phosphoribosylformylglycinamidine synthase	−2.16	5.98E−06
SMU_0191c	Phage-related integrase	−2.35	1.35E−05
SMU_0193c	Conserved hypothetical protein	−2.71	1.92E−05
SMU_0194c	Conserved hypothetical protein, phage-related	−2.71	1.39E−06
SMU_0195c	Hypothetical protein	−2.66	2.53E−05
SMU_0196c	Immunogenic secreted protein (transfer protein)	−2.44	2.55E−05
SMU_0197c	Hypothetical protein	−2.59	2.44E−05
SMU_0198c	Conjugative transposon protein	−2.78	2.31E−05
SMU_0199c	Hypothetical protein	−2.81	1.09E−05
SMU_0200c	Hypothetical protein	−2.68	3.57E−05
SMU_0201c	Conserved hypothetical protein	−2.99	6.44E−06
SMU_0202c	Conserved hypothetical protein	−3.11	1.39E−06
SMU_0204c	Hypothetical protein	−3.58	2.68E−06
SMU_0205c	Conserved hypothetical protein	−3.95	2.06E−07
SMU_0206c	Hypothetical protein	−2.48	4.70E−05
SMU_0207c	Transcriptional regulator	−2.70	2.90E−05
SMU_0208c	Conserved hypothetical protein, FtsK/SpoIIIE family	−3.09	9.09E−06
SMU_0209c	Hypothetical protein	−3.18	8.74E−07
SMU_0210c	Hypothetical protein	−2.66	2.61E−05
SMU_0211c	Hypothetical protein	−3.36	2.07E−05
SMU_0212c	Hypothetical protein	−3.83	6.20E−06
SMU_0213c	Hypothetical protein	−5.15	1.30E−06
SMU_0214c	Hypothetical protein	−4.93	2.04E−06
SMU_0215c	Hypothetical protein	−5.06	6.35E−07
SMU_0216c	Hypothetical protein	−4.80	2.06E−06
SMU_0217c	Conserved hypothetical protein	−6.46	2.22E−07
SMU_0218	Transcriptional regulator	−2.19	5.25E−08
SMU_0651c	ABC transporter, substrate-binding protein	−2.03	3.51E−03
SMU_0653c	*tauC*, ABC transporter, permease protein	−2.03	8.83E−04
SMU_0910	*gtfD*, glucosyltransferase-S	−2.71	4.52E−11
SMU_0932	Conserved hypothetical protein	−3.50	1.38E−04
SMU_0933	*atmA*, amino acid substrate-binding protein	−3.12	4.40E−04
SMU_0934	Amino acid ABC transporter, permease protein	−2.96	8.67E−04
SMU_0935	Amino acid ABC transporter, permease protein	−2.92	7.30E−04
SMU_0936	Amino acid ABC transporter, ATP-binding protein	−2.87	6.37E−04
SMU_0961	Macrophage infectivity potentiator-related protein	−3.52	2.72E−07
SMU_0962	*mmgC*, acyl-CoA dehydrogenase	−3.26	1.37E−06
SMU_0992	Hypothetical protein	−2.53	8.40E−10
SMU_1072c	*bar*, acyltransferase	−2.23	3.18E−07
SMU_1284c	Conserved hypothetical protein	−2.03	4.15E−08
SMU_1286c	*blt*, multidrug resistance permease	−2.02	2.76E−08
SMU_1334	*mubP*, phosphopantetheinyl transferase	−2.42	4.37E−09
SMU_1335c	*mubJ*, enoyl-acyl carrier protein reductase	−2.39	3.57E−10
SMU_1336	*mubI*, conserved hypothetical protein	−2.56	2.38E−09
SMU_1337c	*mubM*, alpha/beta superfamily hydrolases	−2.59	1.76E−10
SMU_1338c	*mubZ*, ABC transport macrolide permease	−2.65	8.92E−09
SMU_1339	*mubD*, bacitracin synthetase	−2.61	3.28E−09
SMU_1340	*mubC*, bacitracin synthetase 1	−2.42	3.12E−08
SMU_1341c	mubB, gramicidin S synthase	−2.20	9.82E−08
SMU_1342	*mubA*, bacitracin synthetase	−2.41	2.80E−08
SMU_1343c	*mubH*, polyketide synthase	−2.34	3.92E−07
SMU_1344c	*fabD*, malonyl CoA-acyl carrier protein transacylase	−2.47	9.22E−07
SMU_1345c	*mycA*, peptide synthetase	−2.35	1.57E−06
SMU_1346	*mubT*, thioesterase II-like protein	−2.15	1.21E−05
SMU_1395c	Hypothetical protein	−2.85	2.01E−06
SMU_1895c	Hypothetical protein	−2.51	4.50E−07
SMU_1896c	Hypothetical protein	−2.72	9.12E−09
SMU_1899	ABC transport fragment	−2.45	1.89E−03
SMU_1912c	Hypothetical protein	−2.16	3.89E−04
SMU_2028	*ftf*, fructosyltransferase	−3.02	2.97E−09
SMU_2076c	Hypothetical protein	−2.67	1.63E−07

a CoA, coenzyme A.

The differentially expressed genes were grouped into 11 functional categories (Fig. 1B and C), with genes encoding transport and binding, DNA metabolism, and hypothetical proteins highly represented in the list of upregulated genes. In contrast, genes encoding hypothetical proteins accounted for more than 50% of the downregulated genes followed by genes involved in transport and binding. The genes that were most highly upregulated during growth under Mn-restricted conditions were those of the dual Fe and Mn transporter *sloABC* operon (≥56-fold to 99-fold), a small open reading frame (*smu185*; 71-fold) with the first 18 nucleotides overlapping the *sloC* gene 3′ end, the *sloR* transcriptional repressor (16-fold), and the uncharacterized *smu770c* gene (6-fold) (Table 1; see also Fig. 1B). BLAST search analysis revealed that the protein encoded by *smu770c* belongs to the Nramp-type transport family predicted to function in metal uptake. The Smu770c protein shared 76% identity with S. agalactiae (group B *Streptococcus*) MntH and 60% and 54% identity with E. faecalis MntH1 and MntH2 proteins, respectively. Of note, S. agalactiae MntH and E. faecalis MntH1 and MntH2 have been recently assigned a role in Mn uptake (32, 39). Other genes upregulated in the absence of Mn were several belonging to the CRISPR2-*cas* operon (*smu1753c* to *smu1764c*; >4-fold) as well as 3 of 4 genes of the *smu995* to *smu998* operon (>2-fold), recently shown to code for an Fe transport system (40).

The genes that were found to be most highly repressed when S. mutans was grown under Mn-restricted conditions were a cluster of genes encoding possible conjugative transposon proteins (*smu191c* to *smu217c*; ≥2.4-fold downregulated). Additionally, genes encoding proteins with predicted roles in amino acid transport (*smu932* to *smu936*), purine biosynthesis (*smu29* to *smu32*), fatty acid biosynthesis (*smu1334c* to *smu1338c*), production of antimicrobial compounds (*smu1339c* to *smu1343c*), and sugar transport and metabolism (*ftf*, *smu2028*, *gtfD*, *smu910*) showed decreased levels of expression under Mn-depleted conditions (Table 1).

### SloABC and MntH are the principal manganese transporters in S. mutans.

**FIG 2:**
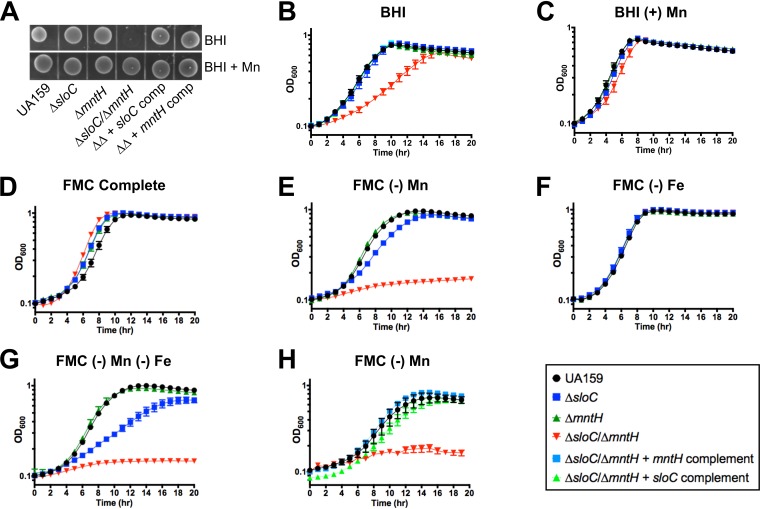
SloABC and MntH promote growth of S. mutans in Mn-depleted environments. (A) Growth of S. mutans UA159 and Δ*sloC*, Δ*mntH*, and Δ*sloC* Δ*mntH* mutant strains along with the double mutant strain complemented with either *sloC* or *mntH* to mid-logarithmic phase (OD_600_ of ∼0.4) on BHI agar. Overnight cultures were spotted onto BHI agar with or without supplementation with 10 μM Mn. Plates were incubated for 48 h before image was obtained. (B to G) Growth of UA159, Δ*sloC*, Δ*mntH*, and Δ*sloC* Δ*mntH* mutant strains in (B) BHI broth, (C) BHI broth supplemented with 75 μM Mn, (D) FMC complete (130 μM Mn), (E) Mn-depleted FMC, (F) Fe-depleted FMC, and (G) Mn- and Fe-depleted FMC. (H) Genetic complementation of the Δ*sloC* Δ*mntH* growth defect in Mn-depleted FMC with either *sloC* or *mntH.* The graphs show averages and standard deviations of results from at least three independent experiments.

Because of the high degree of conservation between Smu770c and previously characterized MntH proteins from other *Firmicutes*, we assigned the name “*mntH*” to the monocistronic transcriptional unit *smu770c*. Here, we sought to characterize the *mntH* gene and investigate the possible cooperative nature of SloABC and MntH in metal acquisition. To accomplish this, we created strains bearing single deletions in *sloC* (Δ*sloC*), which encodes the metal binding lipoprotein of the SloABC system, or in *mntH* (Δ*mntH*), as well as a double mutant strain lacking both *sloC* and *mntH* (Δ*sloC* Δ*mntH*). All mutant strains were initially isolated on brain heart infusion (BHI) agar supplemented with 75 μM Mn. Upon genetic confirmation of the single and double mutants, we tested the ability of these strains to grow in BHI agar and found that the Δ*sloC* Δ*mntH* double mutant was unable to grow on BHI agar without Mn supplementation (Fig. 2A). The Δ*sloC* Δ*mntH* strain was able to grow in BHI broth, albeit at much lower rates than the other strains, reaching similar final growth yields after 16 h (Fig. 2B). Supplementation of BHI agar with 25 μM Mn (BHI+Mn) fully restored the growth defect of the double mutant strain in broth (Fig. 2C). We suspected that the different growth behaviors of the Δ*sloC* Δ*mntH* strain in BHI plates and in broth were due to trace amounts of Mn that had transferred from the overnight BHI inoculum that contained 7 μM Mn. This suspicion was then confirmed by findings showing that the Δ*sloC* Δ*mntH* strain could not grow in unsupplemented BHI agar after a second passage (data not shown). To assess the metal requirements of the mutant strains in a more controlled fashion, growth of the parent UA159 and mutant strains was also monitored in the chemically defined FMC medium (Fe and Mn replete; Table 2) and in FMC medium depleted of Mn (Mn < 90 nM) or Fe (Fe < 90 nM) or both (27). In complete FMC medium, growth of all mutant strains was indistinguishable from that of the parent strain (Fig. 2D). As expected, the Δ*sloC* Δ*mntH* double mutant strain failed to grow in Mn-depleted FMC medium whereas the Δ*sloC* mutant showed a slight growth delay that did not affect the final growth yields (Fig. 2E). Iron depletion alone did not affect growth of the parent strain or of any of the mutant strains, but simultaneous depletion of Fe and Mn exacerbated the slow-growth defect of the Δ*sloC* strain (Fig. 2F and G). Growth of the Δ*sloC* Δ*mntH* strain in plain BHI agar or in Mn-depleted FMC medium was fully restored by complementation when either the *sloC* or *mntH* gene was integrated elsewhere in the chromosome (Fig. 2A and H).

**TABLE 2:** Metal content of media used for growth of S. mutans^*a*^

Metal	Concn (μM)		
	BHI agar	FMC medium	Saliva
Iron	5.91 ± 1.27	82.62 ± 8.8	4.51 ± 0.08
Manganese	0.56 ± 0.27	132.6 ± 14.9	BDL^*b*^
Zinc	10.9 ± 2.01	1.2 ± 0.3	0.4 ± 0.02

a ICP-OES analysis was used to determine the metal content of BHI agar, FMC medium, and pooled human saliva used in this study. Values represent averages and standard deviations of results from at least three independent experiments.

b BDL, below detection limit.

**FIG 3:**
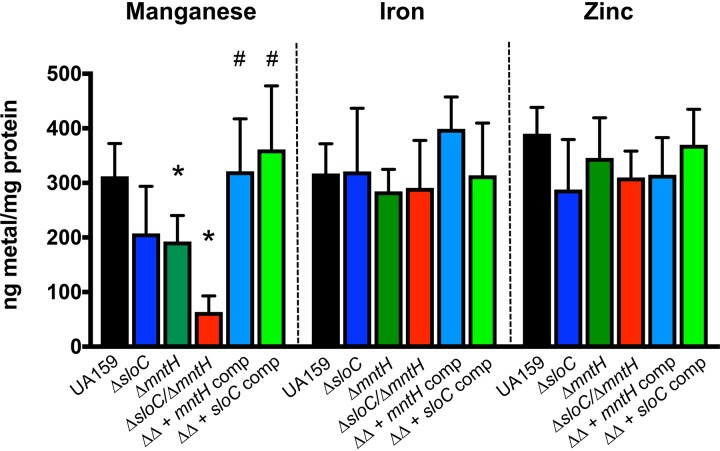
SloABC and MntH are the main Mn transporters in S. mutans UA159. The bar graph indicates the intracellular manganese, iron, and zinc content of S. mutans UA159 and derivatives grown in plain BHI agar to an OD_600_ of ∼0.4. Data represent averages and standard deviations of results from five independent ICP-OES analyses. Student's *t* test was used to compare the metal content of the mutant strains to that of UA159 (*, *P ≤ *0.05) and of the double mutant Δ*sloC* Δ*mntH* (ΔΔ) to that of the complemented strains (#, *P ≤ *0.0005).

Next, we used ICP-OES to determine the cellular metal content of the parent and mutant strains grown to mid-exponential phase in BHI broth (Fig. 3). Despite not showing a growth defect in plain BHI, the Δ*sloC* and Δ*mntH* single mutant strains carried ∼45% less cellular Mn than UA159. In agreement with the results shown in Fig. 2, combined deletion of *sloC* and *mntH* resulted in a more significant (∼80%) reduction in cellular Mn pools. Complementation of strain Δ*sloC* Δ*mntH* with either one of the inactivated genes restored cellular Mn content to parent strain levels. Despite the previously assigned role of SloABC in Fe uptake (38), intracellular quantities of Fe did not differ significantly among the strains. Likewise, no important differences in intracellular zinc content were observed among the strains (Fig. 2). Collectively, these results reveal that SloABC and MntH comprise the principal Mn transport systems of S. mutans, working cooperatively to maintain Mn homeostasis.

### *mntH* is a new member of the SloR regulon.

**FIG 4:**
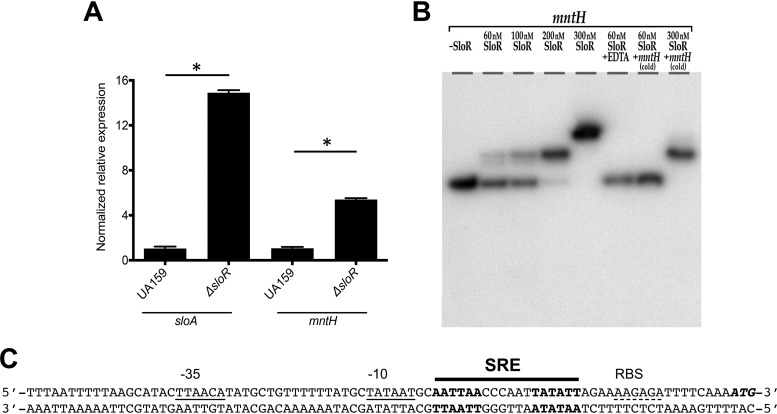
The S. mutans *mntH* gene belongs to the SloR regulon. (A) qRT-PCR analysis indicates that expression levels of *mntH* and *sloA* were upregulated in a Δ*sloR* strain compared to the parent strain UA159. Data represent means ± and standard deviations of results from 3 independent experiments. Student's *t* test was used to compare differences in gene expression between UA159 and Δ*sloR* strains. (B) Regulation of the S. mutans *mntH* gene by SloR is direct. EMSA was performed with a [γ-^32^P]ATP end-labeled *mntH* probe and purified SloR. Reaction mixtures were resolved on 12% nondenaturing polyacrylamide gels and exposed to X-ray film for 24 h at –80°C. The addition of cold competitor DNA (1:1) or 3 mM EDTA in the SloR-*mntH* reaction mixture abrogated the band shift, whereas addition of 300 nM SloR resulted in a supershift. (C) Sequence of the *mntH* regulatory region. The predicted −35 and −10 regions are indicated with a solid underline, and the putative ribosome binding site (RBS) is indicated with a dashed underline. The translational start codon is shown in bold italics, while the predicted SloR recognition element (SRE) containing two hexamers is indicated in bold roman characters.

Transcriptional repression of the *sloABC* operon exerted by SloR has been thoroughly characterized by one of our laboratories (35, 36, 41). A conserved SloR-binding palindrome was identified upstream of the *mntH* gene in one of those studies (36), but the specificity of SloR binding to the *mntH* promoter region was not explored at that time. Here, we used quantitative real-time PCR (qRT-PCR) and an electrophoretic mobility shift assay (EMSA) to determine the SloR-*mntH* relationship. Compared to the parent strain, inactivation of *sloR* (Δ*sloR* strain) resulted in ∼5-fold-increased *mntH* transcription and inactivation of *sloA*, the first gene of the *sloABC* operon, in ∼15-fold-increased transcription (Fig. 4A). In addition, EMSAs revealed that a concentration of as low as 60 nM purified SloR shifted *mntH* probe migration (Fig. 4B) and that the region possibly harbors more than a single SloR binding site given the supershift that was observed with 300 nM SloR. The specificity of SloR binding to the *mntH* probe was confirmed by showing that addition of the metal chelator EDTA or of excess cold probe disrupted the interaction in a concentration-dependent manner (Fig. 4B). The region upstream of the translational start of *mntH* includes a pair of hexamers composing a predicted SloR recognition element (SRE) (Fig. 4C), fitting well with the model of SloR binding that was shown for the S. mutans *sloABC* promoter (42).

### Manganese is critical for S. mutans tolerance of clinically relevant conditions.

**FIG 5:**
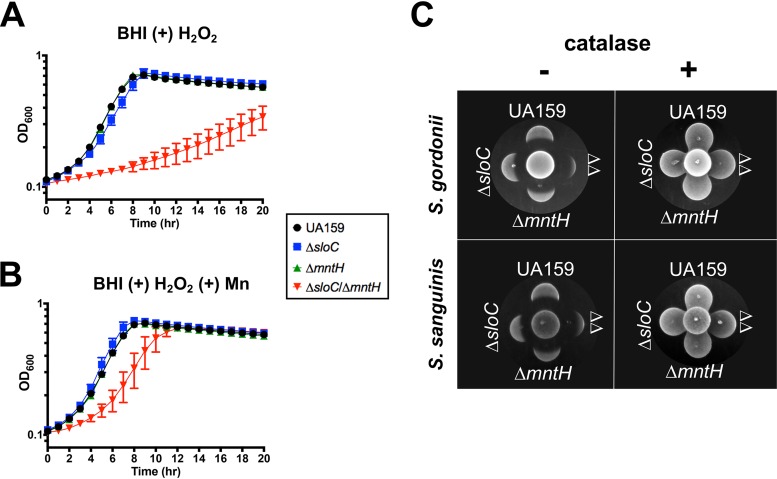
Manganese transport contributes to H_2_O_2_ tolerance. (A and B) Growth of S. mutans UA159, Δ*sloC*, Δ*mntH*, and Δ*sloC* Δ*mntH* strains in the presence of 0.2 mM H_2_O_2_ in (A) plain BHI agar or (B) BHI agar supplemented with 10 μM Mn. (C) A peroxigenic strain (S. gordonii DL-1 or S. sanguinis) SK150 was spotted at the center of a BHI agar plate (supplemented with 2 μM Mn) and grown for 24 h (37°C, 5% CO_2_). S. mutans cultures were then spotted proximal to the peroxigenic strain and grown for an additional 24 h. The center spot of each grouping shown here is the H_2_O_2_-producing strain, while the S. mutans strains are labeled in the figure (ΔΔ corresponds to the Δ*sloC* Δ*mntH* double mutant). As a control, duplicate spotting was performed in which H_2_O_2_ produced by the peroxigenic strains was neutralized by overlaying the inoculum spot with a catalase solution prior to spotting of S. mutans. The images shown are representative of results from three independent experiments.

To examine the importance of Mn in the oxidative stress tolerance of S. mutans, we first grew cells in the presence of a subinhibitory concentration of H_2_O_2_. Under the conditions tested, growth of the parent strain or of the Δ*sloC* strain or Δ*mntH* strain was not affected; however, the growth rates and yields of the Δ*sloC* Δ*mntH* double mutant strain were markedly reduced (Fig. 5A). Importantly, this growth defect was rescued by Mn supplementation (Fig. 5B). In parallel, we tested this same panel of strains in a qualitative competition assay against the net H_2_O_2_-producing oral commensals Streptococcus gordonii and Streptococcus sanguinis. While the antagonizing peroxigenic strain inhibited growth of all S. mutans strains, the growth inhibition of the Δ*sloC* Δ*mntH* strain was much more pronounced (Fig. 5C). The inhibitory effect of the peroxigenic streptococci was abolished by the addition of catalase.

**FIG 6:**
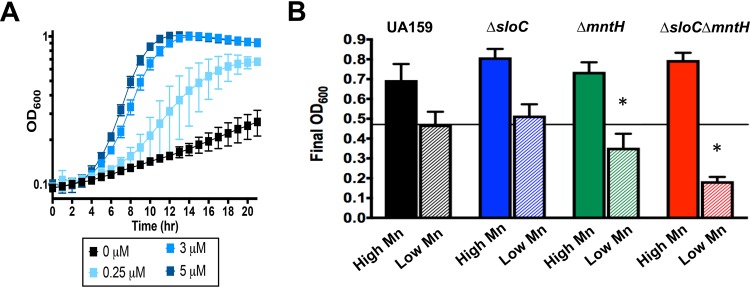
Manganese transport contributes to acid stress tolerance in S. mutans. (A) Growth curves showing the minimal concentration of Mn that fully supports growth of the Δ*sloC* Δ*mntH* strain. The graphs represent averages and standard deviations of results from three independent cultures. (B) Growth of S. mutans UA159, Δ*sloC*, Δ*mntH*, or Δ*sloC* Δ*mntH* in FMC medium adjusted to pH 5.5 containing ∼130 μM Mn (High Mn; solid bars), or 3 μM Mn (low Mn; striped bars). Bars represent means and standard deviations of the final OD_600_ values for five independent experiments. The horizontal line represents the mean final OD_600_ for UA159 grown in FMC medium containing low Mn. Student's *t* test was used to compare the final values determined for the mutant strains to those determined for UA159 grown in the same medium. *, *P < *0.05.

The ability to withstand acid stress is a major virulence attribute of S. mutans that sets it apart as a cariogenic organism compared to the less aciduric commensal streptococci. Recently, the S. agalactiae MntH was shown to play a crucial role in low-pH survival (39). To probe the significance of Mn in acid stress, cultures of parent and mutant strains were grown in FMC medium adjusted to pH 7.0 (control) or pH 5.5 (acid stress) and containing the concentration of Mn indicated in the original recipe (130 μM Mn) or containing the minimal concentration of Mn (3 μM Mn) that sustained optimal growth of the Δ*sloC* Δ*mntH* strain in FMC medium (Fig. 6A). In medium adjusted to pH 7.0, all strains grew well and reached the same final growth yield under conditions of a high or low Mn concentration (data not shown). In medium adjusted to pH 5.5, all strains reached similar final growth yields under the high-Mn condition (130 μM Mn) (Fig. 6B). However, all strains showed reduced final growth yields in the low-Mn medium adjusted to pH 5.5 (compared to high-Mn medium). Moreover, the final growth of the Δ*mntH* and Δ*sloC* Δ*mntH* strains was further impaired in the low-Mn medium adjusted to pH 5.5 (Fig. 6B). Collectively, these results reveal that a minimal threshold of intracellular Mn is a determining factor for the oxidative and acid stress tolerance of S. mutans.

### Manganese promotes sucrose-dependent biofilm formation.

**FIG 7:**
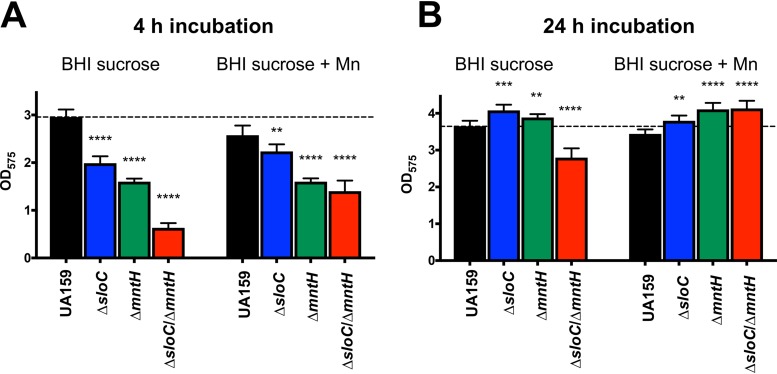
Manganese acquisition is important for sucrose-dependent biofilm formation of S. mutans UA159. Cultures were grown in BHI broth containing 2% sucrose with or without supplementation with 10 μM Mn for 4 or 24 h in saliva-coated microtiter wells. The graph shows averages and standard deviations of results from three independent experiments performed in quadruplicate. **, *P ≤ *0.05; ***, *P ≤ *0.01; ****, *P ≤ *0.005.

Next, we investigated the ability of the Mn transport mutant strains to adhere and form biofilms on saliva-coated microtiter plate wells using BHI medium supplemented with 2% sucrose. In the early stage of biofilm development (4 h of incubation), all mutant strains showed a significant defect in biofilm formation, with the Δ*sloC* Δ*mntH* strain showing the most pronounced defect (∼85% reduction) (Fig. 7A). Supplementation of the growth media with Mn partially restored the early-stage biofilm defect of the double mutant strain (Fig. 7A). After the mature biofilm was formed (24 h of incubation), only the Δ*sloC* Δ*mntH* double mutant continued to show a statistically significant defect in biofilm formation (∼25% reduction); this phenotype was fully restored by Mn supplementation (Fig. 7B). Despite the slow-growth phenotype of the Δ*sloC* Δ*mntH* double mutant in BHI broth (Fig. 2B), no differences in growth (based on optical density at 600 nm [OD_600_] and CFU counts) were observed among strains at the two time points shown in Fig. 7 (data not shown). Collectively, these results support previous observations indicating that the ability to maintain intracellular Mn homeostasis is important for sucrose-dependent biofilm formation.

### Growth and survival of the Δ*sloC* Δ*mntH* strain was impaired in human saliva *ex vivo*.

**FIG 8:**
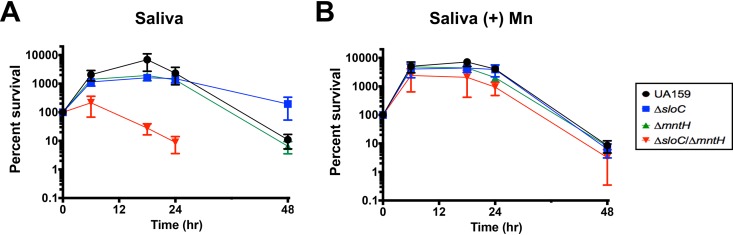
Manganese transport is critical for S. mutans growth and survival in human saliva. Strains (UA159, Δ*sloC*, Δ*mntH*, or Δ*sloC* Δ*mntH*) were grown in plain BHI agar to an OD_600_ of ∼0.3, washed in PBS, and diluted 1:20 in (A) pooled saliva containing 10 mM glucose or (B) pooled saliva supplemented with 10 mM glucose and 10 μM Mn. The graphs show averages and standard deviations of results from four independent experiments.

As a resident of the human oral cavity, S. mutans is bathed in saliva; therefore, the ability to proliferate and survive in this biological fluid is an important aspect of its lifestyle. Here, we tested the ability of parent and mutant strains to grow and survive in pooled human saliva supplemented with 10 mM glucose to promote a more robust level of cell growth. Metal quantifications revealed that our batch of pooled saliva had relatively high Fe (4.51 ± 0.08 μM) and low Zn (0.4 ± 0.02 μM) levels whereas the level of Mn was below the detection limit (Table 2). The parent and single mutant strains grew well in saliva, showing a peak increase in CFU of nearly 2 logs of growth within the initial 18 h, followed by a noticeable loss of cell viability after 48 h (Fig. 8A). On the other hand, the Δ*sloC* Δ*mntH* strain grew poorly within the initial few hours and rapidly lost viability, eventually yielding no viable cells by 48 h. Supplementation of the saliva-glucose media with 10 μM Mn allowed all strains (including Δ*sloC* Δ*mntH*) to reach maximal growth yields faster and to maintain viability comparable to that of the parent strain during the initial 24 h (Fig. 8B).

### SloABC and MntH are required for calprotectin tolerance.

**FIG 9:**
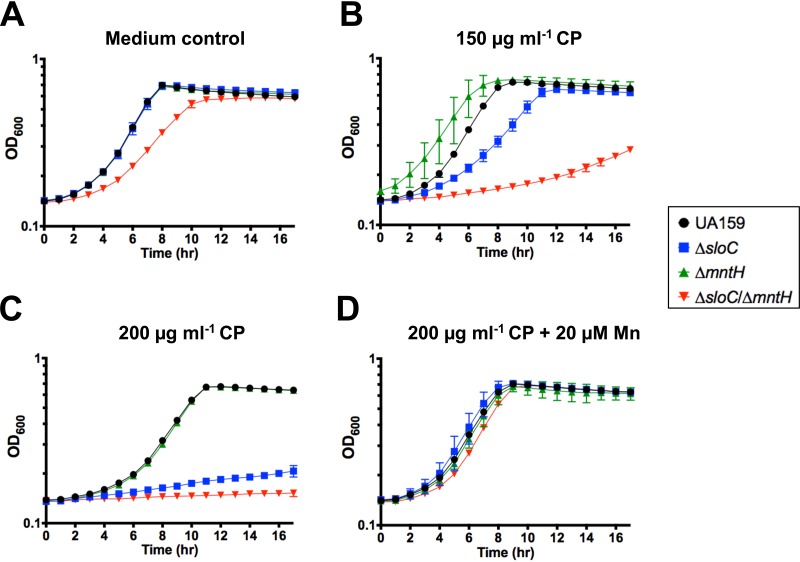
SloABC and MntH are required for S. mutans tolerance of calprotectin. Data represent growth of UA159 and its derivatives in the presence of purified calprotectin (CP). Overnight cultures were diluted 1:20 into BHI agar, grown to early log phase (OD_600_ = 0.25), and then diluted 1:50 in CP medium containing (A) no CP, (B) 150 μg ml^−1^ CP, (C) 200 μg ml^−1^ CP, or (D) 200 μg ml^−1^ CP plus 20 μM Mn. The graphs show averages and standard deviations of results from three independent cultures.

The bioavailability of metals in body fluids is largely dependent on the presence and activity of metal-sequestering proteins such as transferrin, lactoferrin, and calprotectin. In the case of Mn, calprotectin is the major host protein responsible for sequestering Mn (as well as zinc) during infection (3, 6). Recent work revealed that the metal binding properties of calprotectin are more expansive than initially believed, importantly bringing to light the ability of calprotectin to bind to iron *in vivo* (7, 43). Normally found in circulating blood and tissues at low levels, calprotectin accumulates to concentrations of up to 1 mg ml^−1^ in response to inflammation and infection, thereby playing a central role in host-activated nutritional immunity. The apparent ability of calprotectin to scavenge reactive oxygen species adds a further dimension to the relationships among this protein, the host, and the pathogen during infection (6, 44). Here, we tested the ability of S. mutans parent and Mn transport mutants to grow in the presence of subinhibitory concentrations of purified calprotectin (Fig. 9). We found that 150 μg ml^−1^ calprotectin significantly delayed growth of the Δ*sloC* mutant and nearly abolished growth of the Δ*sloC* Δ*mntH* double mutant (Fig. 9B). At 200 μg ml^−1^ of calprotectin, growth of both Δ*sloC* and Δ*sloC* Δ*mntH* strains was fully inhibited (Fig. 9C). In contrast, the parent and Δ*mntH* strains grown in the presence of calprotectin showed an extended lag phase compared to cells grown in calprotectin-free media; that result did not impact final growth yields compared to cells grown under control conditions (Fig. 9A to C). Finally, the growth-inhibitory effect of calprotectin at 200 μg ml^−1^ on the Δ*sloC* and Δ*sloC* Δ*mntH* strains was fully overcome by supplementation with 20 μM Mn (Fig. 9D).

## DISCUSSION

In this study, we showed that Mn is an essential micronutrient for S. mutans and that the ability to maintain Mn homeostasis is important for the expression of virulence factors associated with oral and nonoral infections. Global transcriptional profiling of S. mutans UA159 grown under Mn-depleted conditions led to the identification of a previously uncharacterized Mn transporter, here named MntH, belonging to the Nramp family of transporters. By studying the physiology of the Δ*sloC*, Δ*mntH*, and Δ*sloC* Δ*mntH* strains, we provided unequivocal evidence that SloABC and MntH are the primary Mn transporters in S. mutans and that simultaneous inactivation of *sloC* and *mntH* impaired the fitness of S. mutans under Mn-restricted conditions. However, the Δ*sloC* Δ*mntH* double mutant strain retained the ability to grow under Mn-rich conditions. While the genome of S. mutans does not encode additional transporters with homology to other known manganese transporters, the promiscuous import of metals via noncognate metal transporters and even as part of a complex with another (nonmetal) substrate has been well documented. While evidence showing a major reduction in the levels of intracellular Mn pools and the most severe phenotypes was restricted to the Δ*sloC* Δ*mntH* double mutant, deletion of *sloC* alone significantly impaired growth of S. mutans in the chemically defined media lacking both Mn and Fe as well as in the presence of calprotectin. This finding is in agreement with previous observations made with S. aureus showing that the staphylococcal SloABC homologue, named MntABC, was more important than MntH during infection and that loss of *mntABC* alone resulted in a virulence defect (45). The apparent more prominent role of SloABC than of MntH seen under these specific conditions is likely due to its dual function in Fe and Mn uptake.

Although Nramp-type proteins have been shown to transport different types of trace metal ions such as Fe, Mn, and Zn (46), recent studies performed with S. agalactiae and E. faecalis revealed that the closest homologs of the S. mutans MntH are primarily involved in Mn transport (32, 39). This appears to be the case for S. mutans MntH, as the intracellular levels of Fe or Zn were minimally affected by *mntH* inactivation (Fig. 3). Future studies should include analysis of intracellular metal content from cells grown in media deprived of selected metals to ascertain the specificity of transporters for various metals. Note that while Nramp transporters are commonly found in bacteria, members of this family are absent in some major pathogenic streptococci such as S. pyogenes and S. pneumoniae. On the other hand, all streptococcal genomes encode one copy of an ABC-type Mn transporter homologous to SloABC, though the genetic organizations of the subunits may differ (47). Both of these transporters are known to have multiple membrane-spanning segments. Predictive analysis using TMpred (https://embnet.vital-it.ch/software/TMPRED_form.html) software indicates that the SloB membrane-spanning subunit contains 7 transmembrane helices whereas MntH displays 10 membrane-spanning domains.

In S. mutans, inactivation of *sloABC* resulted in attenuated virulence in a rat model of infectious endocarditis (38) whereas inactivation of the lone Mn transporter in S. pneumoniae abrogated virulence in systemic, respiratory tract, and otitis media infections (33). In E. faecalis OG1RF, which encodes one ABC-type (EfaCBA) and two Nramp-type (MntH1 and MntH2) Mn transporters, inactivation of *efaCBA* and *mntH2* virtually abolished the virulence of E. faecalis in mammalian models (32). In the future, it will be useful to test the virulence potential of the S. mutans Δ*mntH* and Δ*sloC* Δ*mntH* strains in an animal model of infective endocarditis, as we suspect that simultaneous disruption of *mntH* and the *sloABC* operon would abrogate the ability of S. mutans to cause systemic infections, yielding a much more robust phenotype than the single Δ*sloABC* mutant strain displayed (38).

After *sloABC*, *mntH*, and the *sloR* repressor, the next group of overexpressed genes in cells starved for Mn belonged to the CRISPR2 system (∼5-fold average gene upregulation), which is thought to provide sequence-based immunity against “invasion” by mobile genetic elements (48). CRISPRs are often associated with a set of *cas* genes that encode proteins that mediate the defense process. In S. mutans UA159, deletion of the *cas* genes associated with CRISPR2 increased cell sensitivity to heat shock without affecting cell sensitivity to the virulent phage M102 (49). A second CRISPR system present in S. mutans UA159, named CRISPR1, was shown to mediate tolerance toward multiple stresses, including membrane, DNA, and oxidative and heat stress (49). While the mechanism remains to be determined, it seems that CRISPR systems are intimately associated with S. mutans stress responses. Among the genes downregulated under the Mn-depleted condition, 38 genes belong to genomic islands (GI) TnSmu1 (25 genes), a 23-kb region that lies adjacent to a cluster of tRNA genes, and TnSmu2 (13 genes), the largest genomic island found in UA159 (50). While not much is known about the biological roles of these GI in S. mutans, TnSmu2 is responsible for the biosynthesis of a pigment important for oxidative stress tolerance (51). It is also noteworthy that genes belonging to CRISPR systems and to TnSmu1 and TnSmu2 are also differentially expressed in strains lacking the serine protease *clpP*, the transcriptional regulator *covR*, and *cidB* from the Cid/Lrg holin/antiholin system (52–54). Even though ClpP, CovR, and Cid/Lrg modulate diverse biological processes, they seem to share a role in stress tolerance and adaptation. For these reasons, studies to investigate the possible association of these mobile genetic elements with metal homeostasis should be considered in the near future.

SloR was previously shown to repress transcription of the *sloABC* operon in a Mn-dependent fashion by binding to conserved palindromes that define a so-called SloR recognition element (SRE) in the *sloABC* promoter region (35, 36). As a result, growth of S. mutans in Mn-rich media resulted in decreased *sloABC* transcription (36, 38, 55). Previously, a genome-wide characterization of S. mutans UA159 identified a putative SRE in the *mntH* promoter region (29). Here, our results confirm that SloR contributes to the regulation of *mntH*, though the results of both RNAseq and qRT-PCR analyses indicate that SloR repression of the *sloABC* operon is tighter than it is for the *mntH* gene. Such robust *sloABC* repression by SloR can be explained by our previous characterization of cooperative, homodimeric binding between SloR and each of three hexameric repeats that overlap the *sloABC* promoter (42). Whether SloR binding at the *mntH* locus is cooperative and whether the SloR binding sites overlap the *mntH* promoter remain to be determined. While the EMSA results described here support the idea of the presence of two or more SloR binding sites upstream of the *mntH* gene, how this might translate into greater promoter accessibility to RNA polymerase, and thus into more-relaxed *mntH* transcription, warrants further investigation.

The immune protein calprotectin has been shown to play a critical role in hampering the progress of infections associated with pathogens occupying a range of host niches, including Staphylococcus aureus, Helicobacter pylori, Candida albicans, Acinetobacter baumannii, Pseudomonas aeruginosa, and Enterococcus faecalis (32, 56–60). Though earlier reports suggested that calprotectin was incapable of binding Fe in the host environment, new evidence has emerged indicating that calprotectin can starve bacteria for iron in selected media as well as under certain *in vivo* conditions (3, 32, 43, 56–62). Importantly, enzymatic function of the S. mutans superoxide dismutase (SOD) is heavily dependent on Mn for protection from oxidative stresses. Though the enzyme is cambialistic (capable of using either Mn or Fe), studies have shown that the Mn-bound SOD is much more active than Fe-bound SOD (63). Evidence has suggested that restriction of the Mn-dependent SOD by metal sequestration is an important aspect of the contribution of calprotectin to nutritional immunity for S. aureus pathogenesis and that the staphylococcal MntH and MntABC manganese transporters are critical for infection (45, 58).

Previous epidemiological studies have associated high availability of trace metal in the oral cavity with a higher caries incidence in predetermined populations (12–16). In particular, Mn appears to play a prominent role in host-pathogen interactions by serving as a cofactor for bacterial enzymes involved in general metabolism, DNA replication, and oxidative stress tolerance (28). The association of Mn levels with the physiology and cariogenicity of oral streptococci was first examined in the late 1960s and became the subject of more-intensive investigations from the mid-1980s until the early 1990s. Collectively, studies have shown that Mn (i) is an essential cofactor for both cariogenic and noncariogenic streptococci, (ii) plays a major role in the growth of S. mutans at elevated oxygen levels by serving as a cofactor of the superoxide dismutase enzyme, (iii) modulates dextran-mediated aggregation in different species of oral streptococci, and (iv) stimulates carbohydrate metabolism and IPS accumulation in S. mutans (17, 18, 21, 22, 24, 26, 64). Most notably, when added to drinking water, Mn resulted in a significant increase in the total number of carious lesions as well as caries severity in germfree WAGG rats (21). Despite the important advances enabled by those studies, most were conducted prior to or in the early days of the genomic era, when the currently available tools for molecular genetic manipulations and comparative genomics were under development. Here, taking advantage of the contemporary tools available, we confirmed some of those initial discoveries and further expanded our understanding of how Mn influences the pathophysiology of S. mutans. In this report, we confirmed or showed for the first time that some of the major cariogenic traits of S. mutans, such as acid and oxidative stress tolerance, survival in saliva, and sucrose-dependent biofilm formation, are in fact dependent on the intracellular levels of Mn. Further, we have demonstrated that manganese transporters are critical to the ability of S. mutans to tolerate the host immune protein calprotectin, which pathogens encounter in the oral cavity and, particularly, in the bloodstream. These results suggest that strategies to deprive S. mutans of Mn hold great promise in our efforts to combat this important pathogen.

## MATERIALS AND METHODS

### Bacterial strains and growth conditions.

The bacterial strains used in this study are listed in Table 3. S. mutans UA159 and its derivatives were routinely grown in BHI agar supplemented with 75 μM MnSO_4_ at 37°C under anaerobic conditions. For physiologic analyses, bacterial inocula were prepared from overnight cultures grown in BHI medium supplemented with 7 μM MnSO_4_ (BHI+Mn), subcultured 1:20 in plain BHI medium (without Mn supplementation), and grown to the early logarithmic phase (OD_600_ = 0.25) at 37°C in a 5% CO_2_ atmosphere. To assess the ability of S. mutans strains to grow in BHI medium or the chemically defined FMC medium (65), cultures prepared as indicated above were diluted 1:50 into the appropriate medium in a microtiter plate with an overlay of sterile mineral oil to minimize the deleterious effects of oxygen metabolism. Growth was monitored using a BioScreen C growth reader (Oy Growth Curves) at 37°C. Growth in the presence of calprotectin requires the use of 38% bacterial medium and 62% CP buffer (20 mM Tris [pH 7.5], 100 mM NaCl, 3 mM CaCl_2_, 5 mM β-mercaptoethanol). To promote the growth of S. mutans in the CP medium, 3×-concentrated BHI medium was used in combination with the CP buffer. For RNA-Seq analysis, three replicate cultures of UA159 were grown overnight in plain BHI medium as described above and then subcultured 1:20 in complete FMC medium (containing 130 μM Mn) as a control or in Mn-depleted FMC medium in which Mn was omitted from the recipe. Cultures were grown to an OD_600_ of 0.4, harvested by centrifugation, and the bacterial pellets were resuspended in 1 ml RNA Protect bacterial reagent (Qiagen). Following another centrifugation cycle, the supernatants were discarded and the pellets stored at –80°C until use.

**TABLE 3:** Bacterial strains used in this study

Strains	Relevant genotype	Source or reference
S. mutans UA159	Parent, serotype *c*	Laboratory stock
S. mutans UAΔ*sloC*	*smu184*::Spec	This study
S. mutans UAΔ*mntH*	*smu770c*::Erm	This study
S. mutans UAΔ*sloC* Δ*mntH*	*smu184*::Spec, *smu.770c*::Erm	This study
S. mutans GMS584 (Δ*sloR*)	*smu186*::Erm	37
S. mutans Δ*sloC* Δ*mntH*+*sloC*	*sloC* complementation of Δ*sloC* Δ*mntH*	This study
S. mutans Δ*sloC* Δ*mntH+mntH*	*mntH* complementation of Δ*sloC* Δ*mntH*	This study

S. gordonii DL-1	Wild type	Laboratory stock

S. sanguinis SK150	Wild type	Laboratory stock
		
E. coli DH10B	Cloning host	Laboratory stock

### Construction of mutant and complemented strains.

S. mutans strains lacking the *sloC* gene or the *mntH* gene or both were constructed using a PCR ligation mutagenesis approach (66). Briefly, PCR fragments flanking the region to be deleted were ligated to an antibiotic resistance cassette (erythromycin for the Δ*sloC* strain and spectinomycin for the Δ*mntH* strain) and the ligation mixture was used to transform S. mutans UA159 according to an established protocol (66). The double mutant strain was obtained by amplifying the Δ*mntH* region and using the resulting DNA amplicon to transform the Δ*sloC* single mutant strain. Mutant strains were isolated on BHI plates supplemented with 75 μM Mn and the appropriate antibiotic(s). Gene deletions were confirmed by sequencing amplicons containing the antibiotic cassette insertion site and flanking region. The double mutant strain was complemented by cloning the full-length *sloC* or *mntH* gene into the S. mutans integration vector pMC340B (67) to yield plasmid pMC340B-*sloC* or pMC340B-*mntH*. The plasmids were propagated in Escherichia coli DH10B and used to transform the S. mutans Δ*sloC* Δ*mntH* strain for integration at the *mtl* locus. All primers used in this study are listed in Table 4.

**TABLE 4:** Primers used in this study

Primer	Sequence (5′–3′)^*a*^	Application
smu770Arm1F	GGTCTTAGGGACAAGAGTTAAACGC	*mntH* deletion
smu770Arm1R	CCACTGTATTAAC**AAGCTT**CAACTTGC	*mntH* deletion
smu770Arm2F	CCTCGCTGAGT**GAATTC**TTTTTTGG	*mntH* deletion
smu770Arm2R	CTGCAAATTTTAAGACTAACTCTTTTATTGGC	*mntH* deletion
sloCArm1F	GATCACGTTCTGCTTTTG	*slo*C deletion
sloCArm1R	GTAATAATAAGCTTA**GCATGC**TCATTAG	*slo*C deletion
sloCArm2F	GGTTGTTTC**GCATGC**TTCTCTTAAG	*slo*C deletion
sloCArm2R	GATGCTGTTCCATATAC	*slo*C deletion
smu770comp5’	CGG**GGTACC**GAGGATGAAGAGCTTTAATCC	*mntH* complementation
smu770comp3’	CCG**CTCGAG**CCTTCATAGATGAACTTACTGC	*mntH* complementation
sloCcomp5’	CGC**GGATCC**CAGCGGGTTCAAGCATTGTCTTA	*sloC* complementation
sloCcomp3’	CCG**CTCGAG**GGGAGTAAGCGGAAACCTTTCC	*sloC* complementation
sloA.qRT.F	CGTATGCTCTTGGCTCGTTG	qRT-PCR
sloA.qRT.R	ACTCCCATCTCAGTTACACCCT	qRT-PCR
mntH.qRT.F	AATGCCCAGTTTACCAGCCA	qRT-PCR
mntH.qRT.R	TCAGCGAGGTCAATCAGAGC	qRT-PCR
mntH_EMSA_F	CTTTTCGCAATCTGATTGTTTAG	EMSA
mntH_EMSA_R	CATTTTGAAAATCTCTTTTCTAATATAATTG	EMSA

a Restriction sites used to facilitate cloning are indicated in bold.

### RNA analysis.

Total RNA was isolated from homogenized S. mutans cell lysates by acid-phenol-chloroform extractions as previously described (68). The RNA was precipitated with ice-cold isopropanol and 3 M sodium acetate (pH 5) at 4°C before RNA pellets were resuspended in nuclease-free H_2_O and treated with DNase I (Ambion) for 30 min at 37°C. Then, 100 μg of RNA per sample was purified using an RNeasy kit (Qiagen) including a second on-column DNase digestion according to the manufacturer’s instructions. Sample quality and quantity were assessed on an Agilent 2100 Bioanalyzer at the University of Florida Interdisciplinary Center for Biotechnology Research (UF-ICBR). RNA (5 μg per sample) was subjected to two rounds of mRNA enrichment using a MICROBExpress bacterial mRNA purification kit (Thermo Fisher). cDNA libraries with unique barcodes were generated from 100 ng enriched mRNA using an NEB Next UltraII Directional RNA Library Prep kit for Illumina (New England Biolabs). The individual cDNA libraries were assessed for quality and quantity by Qubit. The cDNA libraries were then diluted to 10 nM each, and equimolar amounts were pooled together. The pooled libraries were subjected to RNA deep sequencing (RNA-Seq) at the UF-ICBR using an Illumina NextSeq 500 platform. Read mapping was performed on a Galaxy server hosted by the University of Florida Research Computer using Map with Bowtie for Illumina and the S. mutans UA159 genome (GenBank accession no. NC_004350.2) as a reference. The reads per open reading frame were tabulated with htseq-count. Final comparisons between the control and Mn-depleted conditions were performed with Degust (http://degust.erc.monash.edu/), with a false-discovery rate (FDR) of 0.05 and a 2-fold change cutoff. Quantifications of *mntH* and *sloA* mRNA were obtained by quantitative real-time PCR (qRT-PCR) using gene-specific primers (Table 4) on triplicate samples of the S. mutans UA159 and GMS584 (Δ*sloR*) strains grown to mid-logarithmic phase (OD_600_ of 0.5) according to established protocols (42). Student's *t* test was applied to the analysis of the qRT-PCR results.

### ICP-OES analysis.

The total metal content within bacterial cells was determined using ICP-OES performed at the University of Florida Institute of Food and Agricultural Sciences (UF-IFAS) Analytical Services Laboratories. Briefly, cultures (250 ml) were grown in plain BHI medium to mid-exponential phase (OD_600_ = 0.4), harvested by centrifugation at 4°C for 15 min at 4,000 rpm, and washed first in phosphate-buffered saline (PBS) supplemented with 0.2 mM EDTA to chelate extracellular divalent cations followed by a wash in PBS alone. The bacterial pellets were resuspended in 2 ml 35% HNO_3_ and digested at 90°C for 1 h in a high-density polyethylene scintillation vial. The digested bacteria were diluted 1:10 in reagent-grade H_2_O prior to ICP-OES metal analysis. The metal composition was quantified using a 5300DV ICP atomic emission spectrometer (PerkinElmer), and concentrations were determined by comparisons to a standard curve. Metal concentrations were then normalized to total protein content as determined by the bicinchoninic acid (BCA) assay (Pierce).

### Growth antagonism assay.

The ability of S. gordonii or S. sanguinis to inhibit the growth of S. mutans via H_2_O_2_ production was assessed as described previously (69, 70). Briefly, 8 μl of an overnight culture of S. gordonii DL-1 or S. sanguinis SK150 was spotted in the center of a BHI+Mn agar plate and incubated at 37°C and 5% CO_2_. After 24 h incubation, 8 μl of S. mutans overnight cultures grown in BHI+Mn were spotted near the peroxigenic strain and were similarly allowed to incubate overnight before monitoring for proximal growth defects was performed. To confirm that growth inhibition was due to H_2_O_2_ production, a control condition was included in which 8 μl of 1 mg ml^−1^ catalase solution was spotted on top of the peroxigenic strain spot prior to spotting the S. mutans culture.

### Growth and survival in human saliva.

To test the ability of the S. mutans strains to proliferate and survive in saliva, pooled human saliva was subjected to filter sterilization using a 0.2-μm-pore-size membrane and heat inactivation at 65°C for 30 min. Cultures of S. mutans grown in BHI medium to an OD_600_ of 0.25 as described above were then diluted 1:20 into filtered saliva supplemented either with 10 mM glucose or with 10 mM glucose and 10 μM MnSO_4_ prior to incubation at 37°C in a 5% CO_2_ atmosphere. Immediately upon dilution in saliva and at selected time intervals, 10-fold serial dilutions were prepared in sterile PBS and plated onto BHI+Mn agar for viable plate counting. Saliva samples were collected after obtaining written consent per the study approval from the University of Florida Internal Review Board (Protocol 201600877).

### Biofilm assay.

The ability of S. mutans strains to form biofilms on saliva-coated wells of polystyrene microtiter plates was assessed by growing cells in BHI medium supplemented with 1% sucrose with or without 10 μM of Mn. The wells of the plates were first coated for 30 min with 100 μl of sterile clarified and pooled human saliva. Next, strains grown in BHI+Mn to an OD_600_ of 0.5 were diluted 1:100 in BHI medium containing 1% sucrose and were added to the wells of the microtiter plate. Plates were incubated at 37°C in a 5% CO_2_ atmosphere for 4 and 24 h. After incubation, plates were washed twice with water to remove planktonic and loosely bound bacteria, and adherent cells were stained with 0.1% crystal violet for 15 min. The bound dye was eluted with 33% acetic acid solution, and biofilm formation was then quantified by measuring the optical density of the solution at 575 nm.

### Electrophoretic mobility shift assays.

EMSAs were performed according to established protocols (42). Briefly, primers were designed to amplify the promoter regions of the S. mutans *mntH* gene (Table 4). The resulting amplicons were subjected to end labeling with [γ-^32^P]dATP (Perkin-Elmer) in the presence of T4 polynucleotide kinase (New England BioLabs), after which they were centrifuged through a TE Select-D G-25 spin column (Roche Applied Science) to remove unincorporated [^32^P]dATP. Binding reactions were prepared using 16-μl reaction mixtures containing 1 μl (∼13.25 ng) of end-labeled amplicon, purified native SloR protein at concentrations ranging from 0 to 400 nM, and 3.2 μl of 5× binding buffer (42 mM NaH_2_PO_4_, 58 mM Na_2_HPO_4_, 250 mM NaCl, 25 mM MgCl_2_, 50 mg ml^−1^ bovine serum albumin, 1 mg sonicated salmon sperm DNA, 50% glycerol, 37.5 M MnCl_2_). Samples were loaded onto 12% nondenaturing polyacrylamide gels and resolved at 300 V for 1.5 h. Gels were exposed to Kodak BioMax film for 24 h at 80°C in the presence of an intensifying screen prior to autoradiography.

### Data availability.

Gene expression data have been deposited in the NCBI Gene Expression Omnibus (GEO) database (https://www.ncbi.nlm.nih.gov/geo) under GEO Series accession number GSE139093.
